# Occurrence of multidrug resistant *Escherichia coli* in groundwater of Brij region (Uttar Pradesh) and its public health implications

**DOI:** 10.14202/vetworld.2017.293-301

**Published:** 2017-03-10

**Authors:** Barkha Sharma, Parul, A. K. Verma, Udit Jain, Janaradan K. Yadav, Ravneet Singh, Raghvendra Mishra

**Affiliations:** 1 Department of Epidemiology and Preventive Veterinary Medicine, College of Veterinary Science and Animal Husbandry, UP Pt Deen Dayal Upadhyay Veterinary University, Mathura - 281 001, Uttar Pradesh, India; 2 Department of Veterinary Public Health, College of Veterinary Science and Animal Husbandry, UP Pt Deen Dayal Upadhyay Veterinary University, Mathura - 281 001, Uttar Pradesh, India

**Keywords:** groundwater, microbial analysis, multidrug resistant *Escherichia coli*, physicochemical analysis

## Abstract

**Aim:**

The study evaluates the microbial as well as physicochemical pollution of groundwater of Brij region of Uttar Pradesh, a major tourist destination in the country along with estimating the drug resistance evident in the isolated *Escherichia coli*.

**Materials and Methods:**

A total of 60 samples of groundwater were collected from six different sites and assessed for physicochemical (pH, color, taste, turbidity, total dissolved solids [TDS], total hardness [TH], chlorides, fluorides, nitrates, and iron) and microbiological parameters (standard plate count [SPC], most probable number test [MPN], *E. coli*).

**Results:**

A majority of the samples were found to be out of the range for most of the parameters except iron. Particularly, high values of TDS (up to 9000 ppm), TH (1500 mg/L), chlorides (3250 mg/L), fluorides (2.5 mg/L), and nitrates (100.2 mg/L) were observed at most of the sites in the region highlighting the fact that groundwater of the area is not potable. Samples were turbid and salty to taste. High SPC values, up to 3500 colony-forming unit/ml and coliforms beyond BIS range were found in 40% samples suggesting gross microbial contamination. Only 2 sites (G3 and G5) had low MPN values. Overall 16 (26.67%) *E. coli* were isolated with 3 (18.75%) producing red colonies on conge red agar, hence supposed to be pathogenic. No *E. coli* O157:H7 was isolated. High antimicrobial resistance was observed against amoxicillin and erythromycin, whereas *E. coli* isolates were sensitive toward cefotaxime-clavulanic acid and imipenem. 12 isolates (75%) were multidrug resistant (MDR) with MDR index >20%, and 2 isolates (12.5%) were found to be extended spectrum beta-lactamases positive.

**Conclusion:**

Groundwater is considered to be a safe option for potable water but it is obvious from the findings of this study that considerable physicochemical and microbial contamination is there in groundwater samples of Brij region. The occurrence of MDR *E. coli* in these waters is a matter of great public health concern.

## Introduction

Lack of safe drinking water and poor water sanitation is estimated to take a greater human toll than war, terrorism, and weapons of mass destruction combined. Waterborne diseases account for nearly 1.8 billion human deaths annually and 4.1% of the total disability-adjusted life years global burden of disease with 88% of it being attributable to unsafe water supply, sanitation and hygiene [1]. In 2011, around 768 million people in the third world countries of Asia and Africa relied on unsatisfactory water supply having high levels of pathogen contamination [2]. Not only developing countries but developed nations also see significant outbreaks of waterborne intestinal disease [3].

In many areas, groundwater (well water, submersible water, and handpump) remains the major source of drinking water for people as it is considerably free from impurities and less susceptible to contamination compared to surface water bodies. However, due to a variety of unscientific land and water-based human activities, development and over-exploitation, ground water quality is slowly but surely declining everywhere [4] causing either acute or chronic health effects [5]. Ground water pollution is difficult to detect, and the solutions are expensive, difficult to determine, hard to resolve, tedious and not always effective.

Monitoring the microbiological quality of drinking water relies mainly on the examination of indicator bacteria such as coliforms. Being prevalent in human and animal feces, *E. coli* appears to provide the best bacterial indication of fecal contamination in drinking water as it can be detected with the help of comparatively faster, sensitive, and affordable methods. Its presence in food or water strongly indicates the increased risk of the presence of foodborne pathogenic microbes [6]. Although *E. coli* is commensals of human intestinal tract, several *E. coli* clones have acquired specific virulence factors encoded by mobile genetic elements often located in the chromosomes on pathogenicity islands, not found in commensals [7-10]. Pathogenic *E. coli* strains generally cause three clinical syndromes: Enteric/diarrheal disease, urinary tract infection, and sepsis/meningitis [11].

Antibiotic resistance against commonly used antibacterial agents is increasing due to the non-judicious use of antibiotics in human medicine, agriculture and veterinary fields, especially against beta-lactam drugs. Gram-negative bacteria are most prone as a result of their production of beta-lactamases. Extended spectrum beta-lactamases (ESBLs) have been generally defined as transmissible beta-lactamases that can be inhibited by clavulanic acid, tazobactum, or sulbactum and encoded by genes exchangeable between bacteria. There are more than 400 ESBLs described, clustered into nine different structural and evolutionary families derived from the mutants of temoneira and sulfhydryl variable enzymes and cefotaximase (type lactamases originating from beta-lactamases present in the genus “Kluyvera”) [12].

Brij region (Mathura, Agra and surrounding areas) of Uttar Pradesh is a major tourist destination in India with lakhs of both foreign and domestic tourists visiting these places. At any given time, the floating population is much more than the actual people residing in this area. This creates added burden on the available water resources in the region which already faces considerable water problems. The majority of the population depends on groundwater sources for domestic and agricultural needs. Thus, a study to assess the fecal as well as physicochemical contamination level in groundwater of the region is the need of the hour.

## Materials and Methods

### Ethical approval

No ethical approval was required as no live animals were used in this study. The study was done on water samples collected as per the standard collection methods.

### Sampling site

The present investigation was conducted in Brij region comprising the holy city of Mathura and the city of Taj Mahal, Agra. Mathura district is located on the latitudes 27.41° North and longitudes 77.41° East with an average population of 2,541,894, situated at altitude of 287 m having an area of 3329.4 km. Agra is situated between 27.11° latitude North and 78.0-78.2° longitude East on the bank of Yamuna river. It is situated at an altitude of 171 m above the sea level and has a population of 1,760,285 approximately.

### Sampling

To assess the groundwater quality, a total of 60 water samples from handpumps/borewells/submersibles/wells were collected from six sites, viz., G1 (Maath/Raya), G2 (Goverdhan/Barsana/Vrindavan), G3 (Agra), G4 (Mathura), G5 (Baldeo), and G6 (Refinery and nearby areas), (n=10 for each) from December 2013 to December 2015. For physicochemical analysis, the water samples were collected in 1 L polypropylene bottles and in 500 ml sterile autoclaved glass bottles (Axiva) for microbiological analysis, taking all hygienic precautions, brought to the laboratory on ice and processed within 4-6 h [13].

### Physicochemical analysis

The pH of the water samples was determined by the Hi Media pH indicator paper strip (Hi Media, Mumbai) at the site of sample collection. The taste was analyzed by a panel of people at the Department of VPH, C.V.Ss, DUVASU, Mathura. Turbidity analyzed by water field test kit (multiparameter) technology (High Media, Mumbai), and total dissolved solids (TDS) were determined by water and soil analysis kit (Model No. LT-61-Scientech Lab. Pvt. Ltd., Delhi). Titration method was used to determine total hardness (TH). The chloride, fluorides, nitrate, and iron content were estimated using the Octo Aqua Test kit WT023 (Hi Media, Mumbai).

### Standard plate count (SPC) and most probable number (MPN) test

It was performed as per the method of Edward and Ewing [14] and Cruickshank *et al*. [15]. Water samples were thoroughly shaken, and each sample was analyzed in triplicate after making appropriate dilutions using NaCl solution (0.85 g/L). With a sterile pipette, 1 ml of each dilution and undiluted sample was transferred to sterile Petri plates. Molten and cooled nutrient agar (at 44-44.5°C) was poured into each Petri plate. The plates were rotated gently to mix the medium and the sample thoroughly. Duplicate plates were made for each dilution. The agar was allowed to solidify for 15-20 min and then placed in incubator at 37°C for 48 h. After the stipulated time, colony-forming units (CFUs) in the plates were counted with the help of digital colony counter (Scientech, India). Average of duplicate plates was taken and multiplied with the reciprocal of the dilution used, which gave SPC as number of bacterial CFU/ml of the water sample.

CFU/ml = Average number of colonies counted × Reciprocal of the dilution factor

The MPN test was performed using five tubes method as per the protocol and the results were interpreted using statistical table [16]. The water samples in which there was no acid and gas formation were recorded as negative for MPN.

### Isolation of *E. coli*

All the 60 water samples of ground water were processed for isolation of *E. coli* within 4-6 h of sampling according to Sojka [17] and Edward and Ewing [14]. Enrichment was done in modified trypticase soya agar broth supplemented with 10 mg/L of acriflavin and later streaked onto MacConkey lactose agar plates and incubated at 37°C for 24 h. Lactose fermenting pink, smooth round colonies were then streaked onto eosin methylene blue agar plates (selective plating) and incubated at 37°C for 24 h. Clear blackish colonies with unmistakable greenish metallic sheen were tentatively considered to be *E. coli*, further confirmed by morphological (gram staining technique) and biochemical characteristics [18,19] (Hi Media Rapid Biochemical Identification Kit). All the biochemically confirmed *E. coli* isolates were streaked onto MUG-Sorbitol agar (Hi Media) and incubated at 37°C for 24 h. Colonies showing nonfluorescence under ultraviolet rays were tentatively considered to be *E. coli* O157:H7.

### Congo red (CR) dye binding assay for pathogenic *E. coli*

CR dye binding assay was performed as per the method of Berkhoff and Vinal [20]. All the *E. coli* were streaked on CR agar medium comprising trypticase soya agar enriched with 0.05% CR dye and 0.15% bile salts and incubated at 37°C for 24 h. The plates were further incubated at room temperature for additional 48 h and 72 h. The CR-positive isolates produced brick red colonies after 48-72 h on incubating at room temperature, while CR negative did not bind the dye and produced white or gray colored colonies on CR medium.

### Antibiogram of *E. coli* isolates and assessment of ESBL producers

*In vitro*, antibiogram of all the 16 *E. coli* isolates was performed by disc diffusion technique against 20 commonly used antibiotics (Hi Media) in the treatment of animals [21,22], viz., levofloxacin (LE -5 µg), cefixime (CFM- 5 µg), amoxicillin (AMS -30/15 µg), ofloxacin (OF -5 µg), ciprofloxacin (CIP - 30 µg), cotrimoxazole (COT - 23 µg), chloramphenicol (C - 30 µg), ampicillin-salbactum (A/S – 10/10 µg), gentamicin (GEN - 10 µg), cefuroxime (CXM - 30 µg), amoxicillin-clavulnic acid (AMC - 30 µg), trimethoprim (TR - 10 µg), erythromycin (E - 15 µg), amikacin (AK- 10µg), enrofloxacin (EX - 10 µg), norfloxacin (NX - 10 µg), streptomycin (S - 10 µg), imipenem (IE - 10/750 µg), cefoperazone (CPZ -75 µg) andcefotaxime/clavulanic acid (CEC- 30 µg). A loopful of pure culture for each test isolate was transferred into a test tube containing 5 ml of nutrient broth medium. The broth culture was incubated at 37°C until light to moderate turbidity develops. Inoculum’s turbidity was compared with that of standard 0.5 McFarland. After incubation, the test isolate culture was spun at 5000 rpm for 5 min, to obtain a pellet which was later dissolved using 1 ml of sterile normal saline solution. Plates of Mueller-Hinton agar were seeded with about 1 ml of inoculums by a sterile swab. Antibiotic discs were placed approximately 2.5 cm apart and pressed down to ensure complete contact with the agar surface. The plates were incubated overnight at 37°C. The zones of inhibition diameter were measured for each antibiotic, initially for quality control strain and then for the test strains. The results were compared with the interpretative chart given by the manufacturer, and the test antibiotic was graded as sensitive, resistant or intermediate for respective antibiotics. ESBL producing *E. coli* isolates were phenotypically characterized by double disc synergy test as per the recommended method [22].

### Statistical analysis

The data were statistically processed and analyzed by one-way ANOVA and mean±significant error using Statistical Package Social Science version 16 software, packaged and developed as per the procedure of Snedechor and Cochran [23]. Duncan’s multiple range tests were determined at 5% and 1% level of significance [24].

## Results

### Physicochemical properties of ground water (n=60)

**Figure-1:**
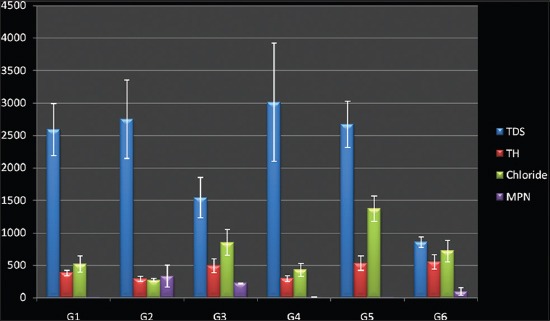
Physicochemical and microbiological parameters of ground water (total dissolved solids, total hardness, chloride, most probable number).

**Figure-2:**
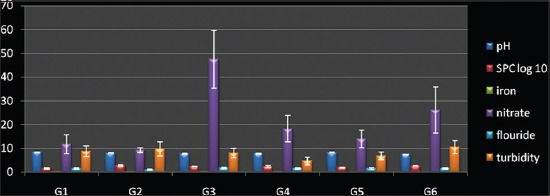
Physico-chemical and microbiological parameters of ground water (pH, standard plate count, iron, nitrate, fluoride, turbidity).

The values of measured physicochemical parameters are shown in Table-1 (Figures-1 and 2). Most of the samples exceeding the limits were alkaline. The highest mean pH was recorded at G3 and the lowest at G1. The mean pH values of G3, G5, and G2 differed highly significantly from pH values of G4, G1, and G6 (p<0.01) (Table-1: Figure-2). All the water samples from G5 and G1 were colorless and odorless; however, none of the samples from any area was sweet enough to be used for drinking purpose as such. In G4 and G6, 40% (4/10) and 60% (6/10) sample had a distinct odor whereas 50% (5/10) samples from G2 were yellowish in color and had a salty odor. There was no significant difference between the turbidity content of water samples from different sites (p>0.05) (Table-1: Figure-2). Considerably high TDS values were recorded with maximum value at 9000 ppm. TDS values of water from G3, G2 and G5 differed significantly from G4 and G6 and G1 (p<0.05) (Table-1: Figure-1). Nine (15%) of the samples exceeded the maximum allowable limit of 600 mg/L of TH set by BIS. There was no significant difference between the values of TH in water samples from all the six areas (p>0.05) (Table-1: Figure-1). The chloride and fluoride levels were overall high with chloride values up to 3250 mg/L from G5 and fluoride up to 2.5 mg/L especially from G6. There was highly significant difference between G3, G4, G2, G1and G6 and G5 (p<0.01) in case of chloride but no significant difference between fluoride content within different areas (p>0.05) (Table-1: Figure-1). The nitrate content was also toward higher side with significant difference present between water samples from G2 and rest of the sites (G1, G3, G4, G5 and G6) (p<0.05) (Table-1). The iron content was within normal range, however, significant difference was observed in iron values between (G2, G6) and (G1, G3, G4 and G5) (p<0.05) (Table-1: Figure-2).

**Table-1:** Physico-chemical properties (pH, turbidity, TDS, TH, chloride, flouride, nitrate and iron) of ground water.

Site	pH			Turbidity (NTU)			TDS (ppm)			TH (mg/L)		
			
	1	2	3	1	2	3	1	2	3	1	2	3
G1	7.6-8.9	40	**8.44^a^±0.13**	0-25.5	50	8.78±2.14	1038-5040	70	**2590^ba^±398**	175-1500	20	382.5±41.67
G2	7.5-8.9	20	**8.14^ba^±0.14**	0-25.5	60	9.75±2.97	850-7500	60	**2750^ba^±606.6**	175-550	0	290±38.94
G3	7.2-8.8	10	**7.78^c^±0.14**	5-25	20	7.93±2.00	800-2690	20	**1540^cb^±315.1**	125-1250	10	490±109
G4	7.1-8.6	10	**7.75^cb^±0.18**	0-10	40	4.70±1.63	900-9000	40	**3010^a^±910.9**	125-550	0	295±47.69
G5	7.6-8.9	40	**8.32^a^±0.14**	0-10	60	6.88±1.59	790-4500	60	**2670^ba^±358.6**	300-1450	30	527.5±111.8
G6	71-8.1	0	**7.47^c^±0.10**	0-25	50	10.48±2.82	720-1350	0	**854c±78.89**	150-1000	20	550±109.9
Overall	7.1-8.9	20	**p=0.000**	0-25.5	75	p=0.541	720-9000	41.6	**p=0.015**	125-1500	15	p=0.106

**Site**	**Chloride (mg/L)**			**Fluoride (mg/L)**			**Nitrate (mg/L)**			**Iron (mg/L)**		
			
	**1**	**2**	**3**	**1**	**2**	**3**	**1**	**2**	**3**	**1**	**2**	**3**

G1	100-1250	10	**515^cb^±126**	0.5-2.5	40	1.56±0.25	0-45.2	10	**11.72±3.95**	0	0	**0.02±0.02**
G2	200-450	0	**272.5±24.28**	0.5-2.5	30	1.17±0.26	0-10.2	0	**9.27±1.03**	0-0.5	10	**0.144±0.05**
G3	200-1000	0	**850±203.3**	0.5-2.5	60	1.78±0.27	10.2-100.2	50	**47.54±12.13**	0-0.3	0	**0.13±0.04**
G4	150-1500	30	**430±98.65**	0.5-2.5	30	1.6±0.2	0-45.9	30	**18.26±5.61**	0	0	**0.06±0.04**
G5	150-3250	70	**1370±193**	0.5-2.5	40	1.46±0.27	10-45	20	**13.92±3.71**	0	0	**0**
G6	100-1700	10	**720±164.6**	0.5-2.5	40	1.47±0.25	0-100	30	**26.02±9.68**	0	0	**0**
Overall	100-3250	20	**p=0.000**	0.5-2.5	40	p=0.673	0-100.2	23.33	**p=0.004**	0-0.5	1.67	**p=0.006**

Mean bearing different superscript in a column differ highly significantly (p<0.01). Means bearing different superscript in a column differ significantly (p<0.05). Figures in bold vary significantly or highly significantly. 1=Recorded range, 2=Percentage of samples out of recommended limit, 3=Mean±SE. SE=Standard error, TDS=Total dissolved solids, TH=Total hardness

### Microbiological qualities of ground water

There was considerable contamination in groundwater of the area with highest SPC value observed to be 3500 CFU/ml and 40 (66.67%) samples exceeding the maximum permissible level (MPL) of <100 CFU/L recommended by BIS. SPC values of samples from (G2 and G4) differed significantly from that of (G1, G6) and (G3, G5) (p<0.05) (Table-2: Figure-2). High coliform contamination was evident by high MPN values up to >1600 coliforms in many samples especially at G2, whereas G3 and G5 had lowest contamination level. A significant difference was observed in MPN values between water samples from (G2, G1, and G6) and (G3, G4, and G5) (p< 0.05) (Table-2: Figure-1). The overall 16 *E. coli* (26.67%) were isolated in all the 60 groundwater samples collected in this study of which only 3 (18.75%) produced red colonies on CR agar indicating their pathogenic nature (Table-2). None of the confirmed *E. coli* isolates produced nonfluorescent colonies on MUG sorbitol agar indicating the absence of O157: H7 serogroup.

**Table-2:** Microbiological properties (SPC, MPN) of groundwater.

Site	Number of samples	SPC			MPN			*E. coli*	Pathogenic *E. coli*
	
		1	2	3	1	2	3		
G1	10	0-500	30	**1.60^c^±0.27**	0-25	10	3.2^b^±2.52	2 (20)	0
G2	10	150-3500	100	**2.83^a^±0.17**	0-1600	70	334.6^a^±169.51	2 (20)	1
G3	10	60-1250	70	**2.34^ba^±0.14**	0-1600	50	221.2^ba^±162.43	3 (30)	1
G4	10	5-3000	70	**2.40^ba^±0.30**	0-25	20	4.9^b^±2.79	0	0
G5	10	30-300	40	**2.0^ca^±0.10**	0-10	-	3.2^b^±1.31	0	0
G6	10	75-2000	90	**2.63^a^±0.13**	0-550	90	95^ba^±57.14	9 (90)	1
overall	60	0-3500	66.67	**P=0.001**	0-1600	40	P=0.094	16 (23.3)	3 (5)

Values in bracket are in percentages. Mean bearing different superscript in a column differ highly significantly (p<0.01). Means bearing different superscript in a column differ significantly (p<0.05). Figures in bold vary significantly or highly significantly. 1=Recorded range, 2=Percentage of samples out of recommended limit, 3=Mean±SE. SPC=Standard plate count, MPN=Most probable number

### Antibiogram of *E. coli* isolates

Antibiogram of all the 16 *E. coli* isolates was performed against 20 most commonly used antimicrobials drugs. Only two *E. coli* (12.5%) were ESBL positive. Overall, the isolates were highly sensitive to cefotaxime-clavulanic acid (81.25%) and imipenem (68.75%) and highly resistant to amoxicillin (75%) and erythromycin (81.25%). Intermediate sensitivity was shown against gentamicin (93.75%), cefperazone (68.75%), levofloxacin (68.75%), ofloxacin (68.75%), norfloxacin and enrofloxacin (68.75%). Of the 16 isolates tested, 12 (75%) were found to be multi-drug resistant (MDR) with an MDR Index (MDRI) >20% including four isolates (25%) having MDRI of >50%. The MDRI was calculated by dividing the number of antibiotics to which isolate was resistant by the total number of antibiotics to which the isolate was exposed (Table-3) [25].

**Table-3:** Occurrence of MDR in *E. coli* isolates.

*E. coli* isolate	Resistance to number of antibiotics out of 20	MDRI (%)	Highly resistant (MDRI>20%)
1	10	50	+
2	6	30	+
3	12	60	+
4	12	60	+
5	6	30	+
6	4	20	−
7	6	30	+
8	5	25	+
9	3	15	−
10	2	10	−
11	9	45	+
12	3	15	−
13	4	20	−
14	3	15	−
15	7	35	+
16	6	37.5	+

MDRI=Multi-drug resistance index, *E. coli=Escherichia coli*

## Discussion

The collected groundwater samples were slightly alkaline (7.1-8.9) in accordance to values recorded in Aligarh (7.31-8.48) by Perween and Fatima [26]. Ashfaq and Ahmad [27] found a pH range of 6.4-7.9 in Agra and 6.3-8.7 in Mathura [28], respectively. Ahmed [29] found pH values in Mathura to be slightly less than the values in this study (7.11-8.11). The quality of groundwater varies from place to place with the depth of water table and is primarily governed by the extent and composition of dissolved solids present in it [16,30]. Exposure to highly alkaline water might cause irritation to eye, skin, and mucous membrane [31]. Acidic pH in well water was reported by Gopinath *et al*. [32] from 10 different locations of Kanakkary Panchayath, Kottayam District, Kerala. The pH of open dug well water in Ethiopia was acidic with 27.3% samples having values below the WHO limit [33]. Most of the samples were free from any disagreeable color but 40% and 68% samples from G4 and G6 had distinct metallic odor. Similar results were obtained by Ahmed [29]. He also found 15% of samples in G1, 50% in G4 and 80% samples from G5 to be sweet in taste, whereas in this study, all the samples were salty to taste. This disparity might be due to different locations or seasons of sample collection in a particular area as values change with respect to geographical and topographical locations or smaller sample size (n=10) in this study. Water gets turbid due to soil runoff, the presence of organic and inorganic matter and prevalence of harmful (pathogenic) microorganisms [34]. In our study, turbidity of 75% samples was more than the desired, hence not suitable for drinking and other purposes. Several authors have found turbidity in this area to be lower than that observed in this study [26-28,16,30]. The mean turbidity values in a study from Nigeria [35] were almost similar to the results obtained in this study. The consumption of highly turbid waters may constitute a health risk as excessive turbidity can protect pathogenic microbes from effect of disinfectants and also stimulate the growth of bacteria during storage [36].

Fluctuations in TDS values are mainly because of dissolved inorganic salts such as carbonates, bicarbonates, phosphates, and sulfates. In Mathura district, water logging and salinity in groundwater have become a universal problem. The general quality of water is brackish with 85% samples having TDS above 1000 mg/L [37]. We found high TDS values similar to the reports of Rawat *et al*. [37] and Tripathi and Thawkar [38], who recorded TDS to be in the range of 570-6692 and 1420-6740 mg/L in Mathura, respectively. Ahmed [29] found TDS values to be lower than this study. Thus, ground water of Mathura is not palatable due to excess salt and mineral content in it and has been shown to increase mortality from all categories of ischemic heart diseases and acute myocardial infarction [39]. The ground water of this region was found to be considerably hard (125-1500 mg/L) with 15% samples exceeding the maximum limit of 600 mg/L of BIS. Lesser values of TH have been recorded in earlier studies from Mathura and Agra [16,27,28,30]. In contrast, very high TH values up to 6250 mg/L in bore well waters in Mathura have also been reported [38].

Chlorides exceeding 250 mg/L impart salty taste to water and cause laxative effects [40]. Chlorides are the most stable components in water and their concentration is largely unaffected by most natural physicochemical and biochemical processes. Hence, the value of its concentration in water is a useful measure to assess water quality [35]. Many studies in Brij region including this study have indicated high chloride levels in the groundwater [28,29,37,38] thereby making treatment of water mandatory before consumption. Fluoride is endemic for Indian subcontinent with 65% of Indian villages in 17 states, being exposed to fluoride risk [41]. These higher fluoride levels in groundwater in Indian continent are associated with igneous and metamorphic rocks [42] and have now become one of the most important toxicological and geoenvironmental issues in Brij region. High values of fluoride have been reported all over India [43,44]. A study by Rawat *et al*. [37] found fluoride level up to 4.6 mg/L in shallow ground water at Shahpur, Mathura, with an average of 3 mg/L in a large part of Mathura. Ahmed [29] recorded a range of 0.5-2 mg/L fluoride content in groundwater of Mathura. Thus, our findings corroborate with these values. Leaching of chemicals, fertilizer, animal manure and pollution from septic and sewage discharges are the main sources of nitrates in water. Although it is considered non-toxic, a high concentration in drinking water is an environmental health concern because it can cause methemoglobinemia or blue baby syndrome in infants, causing death in extreme cases. Nitrate concentration above the MPL of 45 mg/L is reported in 11 states of India, covering 95 districts. Overall nitrate in all groundwater samples in the present study was found to be 0-100.2 mg/L. This finding is in agreement with the observations of Rawat *et al*. [37] who recorded high values of nitrates up to 108 mg/L in groundwater samples from Mathura. In another study indicating groundwater pollution due to urban wastes in Delhi based at Delhi, 57% of samples showed values higher than 45 mg/L with 13% samples exceeding 100 mg/L [45]. These high levels have been attributed to heavy use of nitrogenous fertilizers, cattle waste dumping, waste water disposal, pit latrines, etc., in few agriculturally intensive areas in India [46-49].

The water samples did not have detectable iron content in them. Pandey [30] and Ahmed [29] also found iron to be absent or in trace amounts in ground water samples of Agra and Mathura, respectively. Iron in water can affect the flavor of water and promotes the growth of iron bacteria in water [50]. Similarly, permissible limits of iron were found in water samples in Namdayal rural area of Kurnool district Andhra [51] and well waters of Abokuta, Nigeria [52]. However, iron level in drinking waters of Renigunta near Tirupati, Andhra, ranged from 0.16 to 0.71 mg/L with many samples exceeding the desirable range but lying within the MPL [53].

In this study, values of SPC and MPN of coliform indicated high microbial especially fecal contamination in ground water. Coliform count of 40% samples exceeded the MPL of ≤10 coliforms/100 ml for untreated water. Contamination was evident especially in G1 and G2 sites, whereas none of the samples from G5 were contaminated. Ahmed [29] also found the microbial quality of groundwater of this area to be unsatisfactory. Drinking water from Junagadh, Gujarat, showed heavy presence of coliforms up to >2400/100 ml [54], but the pollution level of groundwater was much less in Bargarh district, Orissa, India [55]. In Hyderabad, water of protected wells had MPN in excess of 800 and was not potable [56]. In Egypt also, well water samples had high level of total coliforms and fecal coliforms [57].

Water is a very efficient vehicle for the dissemination of *E. coli* and has been implicated in various outbreaks worldwide [58-60]. In this study, out of 60 groundwater samples, 16 *E. coli* were isolated (26.7%) indicating fecal contamination of groundwater of the area. Singh [61] who isolated 4 *E. coli* in 16 bore well waters (25%) from Mathura. However lesser load of 9.23% was found by Ahmed [29], in groundwater samples from Mathura but no pathogenic *E. coli* could be isolated. CR assay is used as a phenotypic marker to distinguish between virulent invasive and avirulent *E. coli*. In this study, out of 16 *E. coli*, three (18.75%) were pathogenic as they produced red colonies on CR agar. Berkhoff and Vinal [20] established a direct correlation between ability of clinical isolates of *E. coli* to bind CR dye and their ability to cause septicemic infection in chicken.

Antibiotic resistance in *E*. *coli* has been globally identified in isolates from environmental, animal and human sources [62] which might be a consequence of the non-judicious use of antimicrobials in animals, bringing about chromosomal mutations and hence phenotypic changes [63,64]. Various workers all over the globe have reported the presence of antibiotic resistant *E. coli* in waters [65-69]. In this study, 12 (75%) isolates were found to be highly resistant with a MDRI >20% including 4 isolates (25%) having MDRI of >50%. MDR (3-6 antimicrobials) was seen in 62.96% of *E. coli* isolates from drinking water [70]. The prevalence of multiple-antibiotic-resistant *E. coli* was observed in Dutch surface and waste water samples [71], Portugal fountains [72] and Romanian rivers [73]. A high incidence of ESBL *E. coli* has been reported by Doughari *et al*. [74] and George *et al*. [75] in environmental isolates. Presence and persistence of pathogenic ESBL *E. coli* in surface, ground and drinking water samples, is a matter of great public health concern because water may serve as a potential source of these resistant bacteria to humans.

## Conclusions

From the findings of the study, it is evident that groundwater of Brij region is heavily contaminated with various salts, organic and inorganic impurities. The occurrence of MDR *E. coli* in these groundwater samples is a matter of great public health concern as it might serve as a source of drug resistant *E. coli* to humans and animals which consume these waters apart from other health problems which might be there due to the presence of other impurities. As the region is a prominent tourist destination, this problem becomes graver. Thus, it is suggested that groundwater should be consumed only after proper treatment and a detailed survey should be carried out with larger sample size to assess the real extent of the problem.

## Authors’ Contributions

Parul, AKV, and UJ executed the study design and analyzed the data. RS and RM helped in collection of samples and laboratory testing of them along with analysis of data. BS drafted and revised the manuscript with the help of JKY, AKV, and UJ. All authors read and approved the final manuscript.
